# The Effects of Different Concentrate-to-Forage Ratio Diets on Rumen Bacterial Microbiota and the Structures of Holstein Cows during the Feeding Cycle

**DOI:** 10.3390/ani10060957

**Published:** 2020-05-31

**Authors:** Lijun Wang, Yang Li, Yonggen Zhang, Lihua Wang

**Affiliations:** 1 College of Animal Science and Technology, Qingdao Agricultural University, No. 700 of Changcheng Road, Qingdao 266000, China; 201901173@qau.edu.cn; 2 College of Animal Science and Technology, Northeast Agricultural University, No. 600 of Changjiang Road, Harbin 150030, China; dahai0806702@126.com

**Keywords:** high-throughput sequencing, rumen microbiota, rumen metabolism, feeding cycle, cows

## Abstract

**Simple Summary:**

The rumen is well-known as a natural bioreactor for the highly efficient degradation of fibers, and rumen microbes play an important role in fiber degradation. The rumen is a dynamic system that processes fibrous plant materials, and the rumen microbiota undergoes significant changes during the feeding cycle. However, there are few literatures about the feeding cycle effects on the microbial community. Therefore, we used high-throughput sequencing technology to monitor the ruminal bacterial changes during the feeding cycle. This study showed that there were regular changes in microorganisms and pH, and the relative content of the microorganisms recovered to their previous values prior to the next feeding. The microbial diversity of the forage group was higher than that of the concentrate group during the feeding cycle. At an earlier stage of feeding, the soluble carbohydrates are sufficient for microbial fermentation. Altogether, the results will help us to better understand the ruminal bacterial changes of dairy cows during the feeding cycle under high-forage/concentrate diets, which could provide further explanations of the interactions among rumen microorganisms and help manipulate the rumen metabolism.

**Abstract:**

The objectives of this study were to investigate the ruminal bacterial changes during the feeding cycle. Six ruminally cannulated Holstein cows were used in this experiment. The high-forage (HF) and high-concentrate (HC) diets contained 70% and 30% dietary forage, respectively. Dairy cows were fed their respective diets for at least 28 days, then samples were collected at 0, 2, 4, 9, 12, 16 and 20 h post-feeding. The results showed that pH, the concentration of (total volatile fatty acids) TVFAs and the percentages of acetate, propionate and butyrate were significantly affected by diet and time interactions. The diversity of rumen microbiota in HF dietary treatments was significantly higher than that in the HC dietary treatments. ACE (Abundance-based Coverage Estimator) and Chao 1 indices peak at 12 h post-feeding and then decline over the next 8 h. The rumen microbiota was mainly composed of the phyla Firmicutes, Bacteroidetes and Proteobacteria without considering the diet and time. The Phylogenetic Investigation of Communities by Reconstruction of Unobserved States (PICRUSt) functional profile prediction indicated that the carbohydrate metabolism was different at 9, 12 and 20 h post-feeding time, which revealed that the soluble carbohydrates were enough for microbial fermentation shortly after feeding. This research gave a further explanation of the interactions among rumen microorganisms, which could further help manipulate the rumen metabolism.

## 1. Introduction

In ruminants, the rumen is an anaerobic fermenter for plant fibers degradation, where the structural carbohydrates were converted into soluble carbohydrates, and the energy produced by the rapid fermentation of soluble carbohydrates is used by the ruminants themselves and rumen microorganisms [1]. This process is mainly attributed to rumen microorganisms, which can convert some substances that cannot be used by human into meat and milk [1]. Rumen microbes play an important role in the ruminant digestion of plant fibers, which is the result of millions of years of natural selection and evolution [1,2,3]. However, rumen microorganisms are unstable and exhibit changes with rumen pH, ionic strength, redox potential and fermentation time changes [4].

The rumen environment is affected by many factors, such as diet type, feeding frequency, etc. [5,6,7]. The dietary concentrate-to-forage ratio affects rumen pH, volatile fatty acids, NH_3_-N and rumen microbial flora. Suitable forage-to-concentrate (F:C) ratio diets can provide balanced nutrition for ruminants, improved feed conversions and animal performances and optimized rumen microflora [8]. Most previous research focused on the effects of different dietary F:C ratios on growth performances, carcass characteristics, blood profiles, milk physicochemical characteristics and the related microorganism [9,10,11,12]. Meanwhile, another important factors that changes the structure of the bacteria is probably the feeding cycle, during which the rumen environment undergoes a dynamic turnover—especially, the pH changes [13]. The rumen microorganisms are sensitive to pH changes and exhibit changes with environmental changes [13,14]. How do these microbes change? Although the majority of previous researches have been conducted on different dietary forage-to-concentrate ratios, they only researched the effects of dietary forage-to-concentrate ratios on volatile fatty acid (VFA) contents and a part of microbial and could not monitor the entire microflora.

During the last few decades, high-throughput sequencing can be immediately researched and outputs a lot of raw data. Compared with classical culture-based microbiology methods, using high-throughput sequencing technology can detect the genetic information of almost the entire microorganisms and, more precisely, reflect the microbiome structure changes [7,15].

Additionally, as mentioned above, most research has been conducted on forage-to-concentrates, but a few studies have researched the effects of the feeding cycle on the diversity of the entire microflora in dairy cows. We hypothesized that feeding more forage would increase the microbial diversity and reduce the differences of microorganisms at different time points during the feeding cycle. As thus, this study was designed and carried out to explore the microbial diversity during the feeding cycle under high-forage/concentrate diets and using the high-throughput sequencing technology that provides an abundant knowledge about the bacterial community changes during the feeding cycle.

## 2. Materials and Methods

### 2.1. Ethics Statement

All animal studies were conducted according to the animal care and use guidelines of the Animal Care and Use Committee of Animal Science and Technology College, Qingdao Agricultural University (Qingdao, China), No. SYXK(Lu) 2017 0005.

### 2.2. Animals, Diets and Sample Collection

The experiment was conducted in the Nestle Dairy Farming Institute (Shuangcheng, Harbin, China). Six ruminally cannulated Holstein cows (dry period) of similar age (3.25 ± 0.25, year) and weight (563 ± 22, kg) were randomly assigned into 2 dietary treatments. Cows were housed in tie-stalls bedded with wood shavings and were allowed to exercise daily. The treatments contained forage at 70% (high-forage, HF) and 30% (high-concentrate, HC) of dietary (dry matter basis), respectively. For 4 weeks before sampling, cows were fed once-daily meals at 8:00 h and allowed ad libitum consumption of 110% of their expected intake and had free access to fresh drinking water. The ingredient and nutritional compositions of the two diets are presented in Table S1 (In Supplementary Materials). The ingredient and nutritional compositions of the diets are calculated by the CPM-Dairy v3 (Cornell-Penn-Miner Dairy 3 version).

Rumen content samples were collected before feeding (i.e., at 0 h) and 2, 4, 9, 12, 16 and 20 h post-feeding via a ruminal fistula. Each cycle was divided into two days’ sampling: 0, 4, 9 and 16 h on the first day and 2, 6, 12 and 20 h on the second day. Three consecutive cycles were collected, totaling 6 days. Samples of the same animal and the same time were mixed together. Samples were strained through 4 layers of cheesecloth and analyzed for pH, volatile fatty acids (VFAs), enzymes and 16S rRNA gene sequence microbial community. In particular, the pH of each sample was measured using a portable pH meter (DPH-2; ATAGO, Guangzhou, China). One part of the samples for DNA extraction and enzyme activity determination were snap-frozen in liquid nitrogen and then taken to the laboratory and stored at −80 °C until analysis. The rest of samples were used for the analysis of VFAs. Freshly prepared metaphosphoric acid (25% *w/v*; 1 mL) was added to 5 mL of filtered rumen fluid and stored at −20 °C for the measurement of VFAs.

### 2.3. Ruminal Fermentation Parameters

For the determination of VFAs, samples with metaphosphoric acid were thawed at room temperature and then centrifuged (12,000× *g* for 15 min at 4 °C). The supernatant was used to measure the VFA concentration by gas chromatography (GC-2010 Pro, Kyoto, Japan), the detailed method according to Stewart and Duncan [16]. For the enzyme activity assay, frozen pellets were thawed at room temperature. After being centrifuged at 3000× *g* for 10 min (4 °C), 10–15 mL of supernatant was taken for sonication (power, 400 W; crushed three times for 30 s each time at 30 s intervals), and the crushed liquid was subsequently tested. The assayed CMCase and β-glucosidase activity was measured using the 3,5-dinitrosalicylic acid method [17].

### 2.4. DNA Extraction and 16S rRNA Gene Amplicon Preparation

Genomic DNA was extracted using the Cetyltrimethylammonium Ammonium Bromide (CTAB)-based DNA extraction method and the formula of made necessary reagents, as previously described [18,19]. The final DNA was dissolved in 50 μL of TE buffer and stored at −80 °C. The concentration and purity of DNA was determined by measuring the A260/280 value using a NanoDrop 2000 spectrophotometer (Thermo Fisher Scientific, Waltham, MA, USA), and the integrity checked by agarose (1.5%) gel electrophoresis of each sample.

The hypervariable V3–V4 region of the bacterial 16S rDNA gene was amplified from each DNA sample; the V3–V4 region of the 16SrRNA gene was amplified using the following primers: forward 5’-ACT CCT ACG GGR SGC AGC AG-3’ and reverse 5’-GGA CTA CVV GGG TAT CTA ATC-3’. The PCRs were performed using the Applied Biosystems’ Veriti Thermocycler (Thermo Fisher Scientific Co., Ltd., Shanghai, China) in a 20-mL reaction volume. Thermocycling parameters were as follows: initial denaturation at 95 °C for 2 min; 30 cycles of further denaturation at 95 °C for 15 s, annealing at 50 °C for 30 s and extension at 68 °C for 1 min and a final extension at 68 °C for 7 min. All PCR reactions were performed in triplicate, and products were combined. The integrity of PCR products was checked by gel electrophoresis and purified with the Agarose Gel DNA Extraction Kit (TaKaRa; Dalian, China). The concentrations of PCR products were measured using a NanoDrop 2000 spectrophotometer (Thermo Fisher Scientific, Waltham, MA, USA), then merged according to DNA concentration.

### 2.5. Illumina HiSeq Sequencing and Sequence Analysis

The paired-end sequencing was conducted by an Illumina HiSeq 2500 system. The resulting sequences were then screened and filtered for quality and length. Sequences with short reads were extended by merging paired-end reads using FLASH v1.2.7 [20]. Any read pairs that could not be assembled and any single reads were discarded. Sequences were trimmed, quality-filtered and deconvoluted based on the 12-bp barcode sequence. Chimeras were identified and removed using UCHIME v4.2 to obtain the effective tags [21]. Subsequently, the sequences were processed and analyzed using Quantitative Insights into Microbial Ecology (QIIME, v1.8.0), as described by Caporaso et al. [22]. The high-quality sequences were classified into operational taxonomic units (OTUs) biased on 97% similarity. Representative OTU were classified by Uclust [21] against the Greengenes reference database [23]. Singletons were removed before further analysis [24]. These OTUs were used for microbial diversity and rarefaction curve analysis via MOTHUR software.

### 2.6. Bioinformatics and Statistical Analysis

Alpha diversity indices (i.e., ACE, Chao1, Shannon and Simpson) were calculated by QIIME from rarefied samples using for richness and diversity indices of the bacterial community. Beta diversity was measured on the basis of Bray–Curtis distances, which were calculated by QIIME and displayed using nonmetric multidimensional scaling (NMDS) analysis. The significance of grouping in the NMDS plot was proofed using analysis of similarity (ANOSIM) and was carried through QIIME [25]. Linear discriminant analysis (LDA) effect size (LefSe) analysis was carried to show the differentially abundant feature in the HF dietary treatment and the HC dietary treatment [26]. The absolute LDA (linear discriminant analysis) score was log10 ≥ 3.0. Phylogenetic Investigation of Communities by Reconstruction of Unobserved States (PICRUSt) was used to forecast the carbohydrate metabolism gene content in the rumen microbiota of HF and HC dietary treatments based on systematics acquired from the Greengenes reference database [23,27]. GraphPad Prism (version 5; GraphPad Software, Inc., San Diego, CA, USA) was used for the data analysis and to draw the data charts.

Data of pH (pH value converted to the hydrogen ion, and statistical analysis used the H ions. Then, converted the mean H ions to pH and reported that.), VFAs, OTU, alpha-diversity indices and the dominant genus were constructed using SAS PROC MIXED (SAS 9.4, SAS Institute Inc., Cary, NC, USA). The model included time, feed and time × feed as the fixed effects, treating individual groups as the trial units. A *p*-Value < 0.05 was considered statistically significant, and *p*-Value < 0.01 indicated the differences are extremely significant.

## 3. Results

### 3.1. Dynamic Changes of Rumen Fermentation Characteristics of Cows Fed Two Different Diets

In the beginning, the pH decreased and then gradually increased; the rock bottom of the HC dietary treatment and HF dietary treatment appeared at 4 h and 6 h post-feeding (Table 1); however, the TVFA concentrations showed a reverse pattern (Table 2). With the exception of TVFAs and the proportion of the valerate, a significant (*p* < 0.05) interaction between the feed and time was observed for all fermentation parameters. The concentration of TVFA and the proportion of the propionate, butyrate, valerate and isovalerate were significantly higher in the HC group than those of the HF group. Therefore, the acetate-to-propionate (A:P) ratio was lower (*p* < 0.05) for cows fed the HC treatment than cows fed the HF treatment. This indicated that the fermentation parameters change was significant during the feeding cycle, and HC group increased the content of the TVFA and decreased the pH value.

### 3.2. Sequencing Depth and Rumen Bacteria Diversity of Cows Fed Two Different Diets

**Figure 1:**
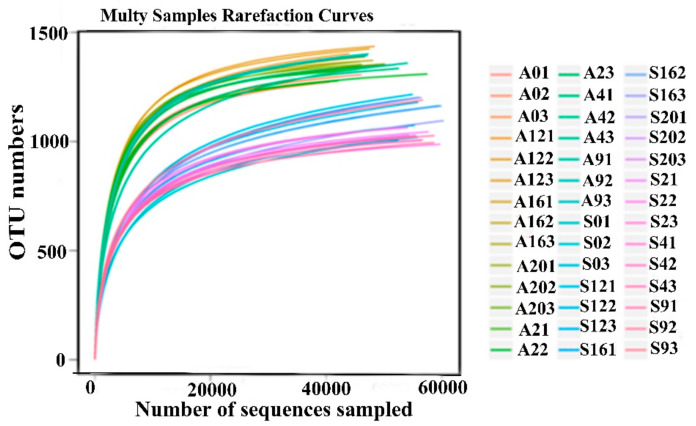
Rarefaction analysis of the different samples. Rarefaction curves of the operational taxonomic units (OTUs) clustered at a 97% sequence identity across the different groups. A represents the high-forage (HF) group samples, and S represents the high-concentrate (HC) group samples. The last number of each sample (1, 2 and 3) represents three repetitions, and the numbers in the front represent the different time points. For example: A01, A = HF group, 0 = 0 h and 1 = first repetition and S01, S = HC group, 0 = 0 h and 1 = first repetition.

The HF and HC groups had an average of 62,741 and 63,344 reads per rumen sample, respectively. The average sequence lengths of the HF and HC groups were 420 and 419 bp, respectively (Table S2 in Supplementary Materials). The rarefaction curves of each sample almost approached the saturation plateau (Figure 1), which showed that the sequence depth of each sample was adequate.

The number of OTUs of the HF dietary treatment was significantly more than that of the HC dietary treatment (Table 3). During a feeding cycle, the OTU declined its minimum just before feeding and increased for approximately 12 h and then gradually decreased to the initial numbers at about 20 h post-feeding. The results of the alpha-diversity indices (Ace, Chao 1, Simpson and Shannon) are shown in Table 3. With the exception of the Shannon indices, a significant (*p* < 0.05) effect by time was observed for the alpha-diversity indices; the Ace and Chao 1 indices reached their maximum values at 12 h post-feeding and then gradually decreased to their initial values at approximately 20 h; however, the Shannon index was higher in the HF treatment than that of the HC treatment. The Simpson index was higher in the HC group than that of the HF group.

**Figure 2:**
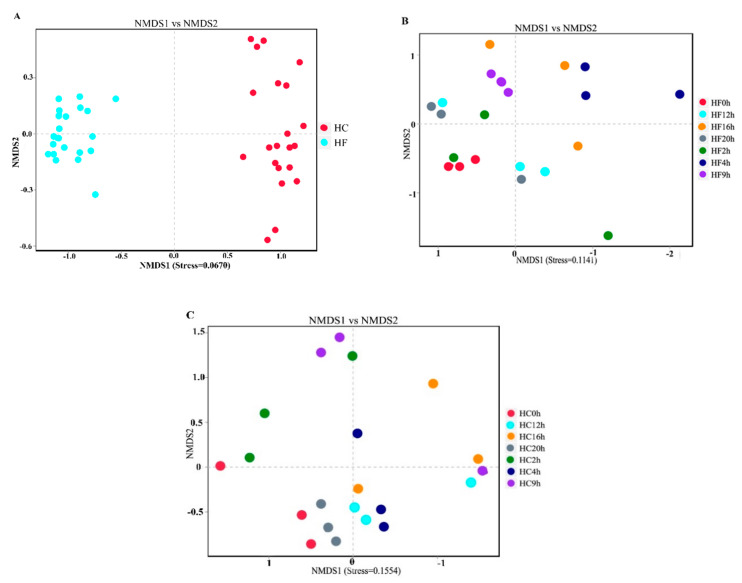
Nonmetric multidimensional scaling (NMDS) analysis. (**A**) HF and HC groups. (**B**) HF group. (**C**) HC group. Each point in the graph represents one sample, and same colors represent same groups. The distance between points represents the level of difference. Stress lower than 0.2 indicates that the NMDS analysis is reliable. The closer the samples in the graph, the higher their similarity.

**Figure 3:**
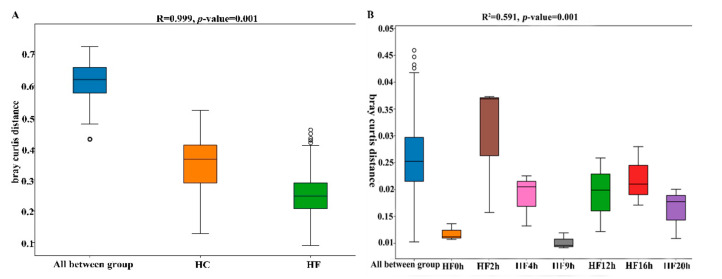

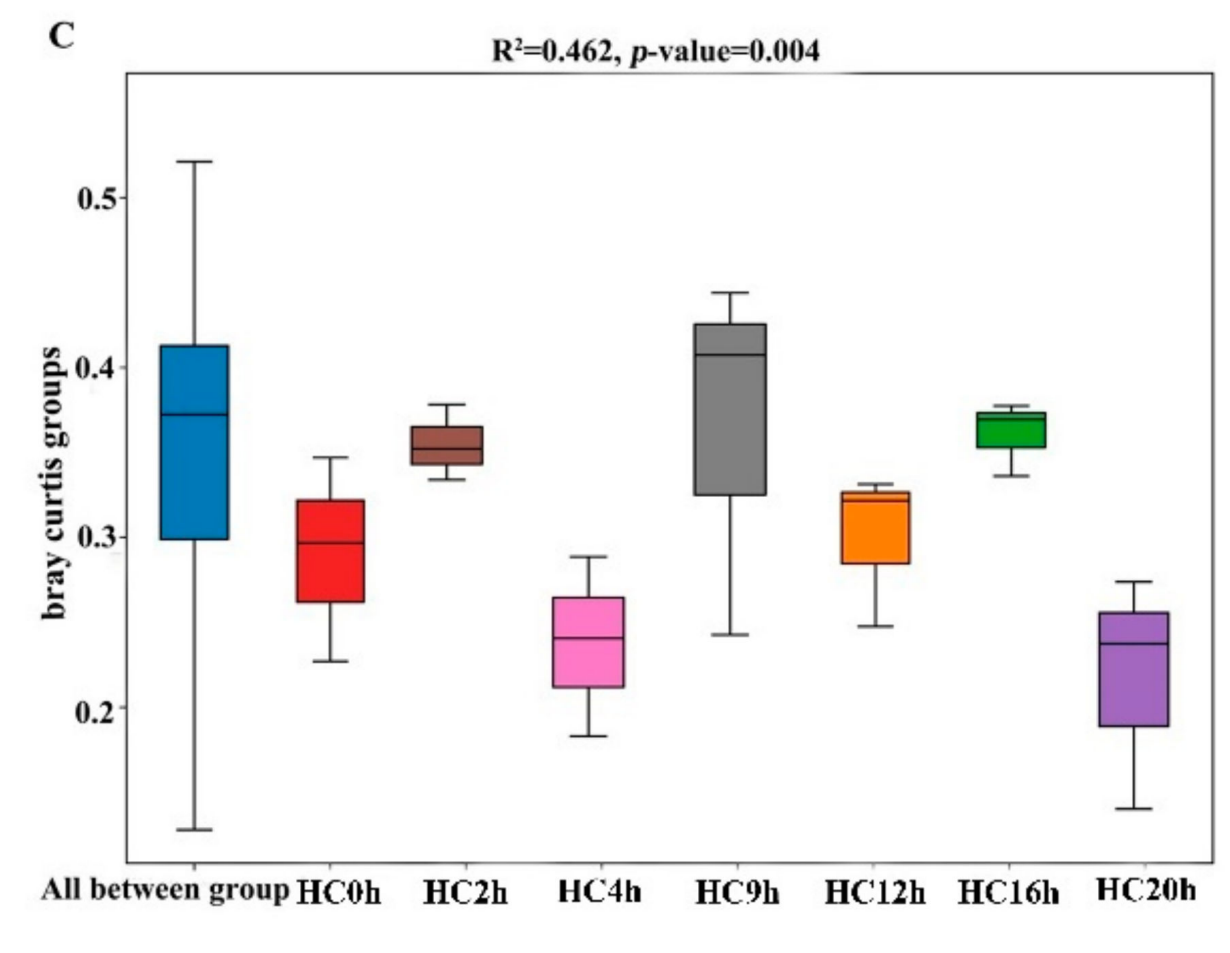
Box plot of inter-group and intra-group beta distance (ANOSIM analysis). (**A**) Beta distance of HF and HC groups. (**B**) Beta distance of HF dietary group. (**C**) Beta distance of HC dietary group. The *x*-axis represents the grouping, and the *y*-axis represents the distance calculated by bray_curtis. The data in the box is the distances of the inter-group and intra-group, respectively. R-value: R-value range (−1, 1). The R-value ≤ 0 represents no significant differences of the inter-group and intra-group, and R-value > 0 shows that inter-group differences are greater than intra-group differences. Boxes represent the interquartile range (IQR) between the first and third quartiles (25th and 75th percentiles, respectively), and the horizontal line inside the box defines the median. Whiskers represent the lowest and highest values within 1.5 times the IQR from the first and third quartiles, respectively.

Beta diversity is used to analyze the temporal and spatial changes in species composition, reflecting whether there is difference in bacterial communities between groups. The NMDS plot showed the dissimilarity of the microbial community and also revealed a distinct structure between two dietary treatments (Figure 2A) and sampling times (Figure 2B: intra-group of the HF dietary treatment and Figure 2C: intra-group of the HC dietary treatment). The box plot showed the beta distance of the inter-group and intra-group (Figure 3A), and the results showed that there were extremely significant differences in the bacterial communities between the HC and HF treatments, the intra-group of the HC dietary treatment (Figure 3B) and the intra-group of the HF dietary treatment (Figure 3C) (*p* < 0.01).

### 3.3. Ruminal Bacteria Changes within the Two Treatments During the Feeding Cycle

**Figure 4:**
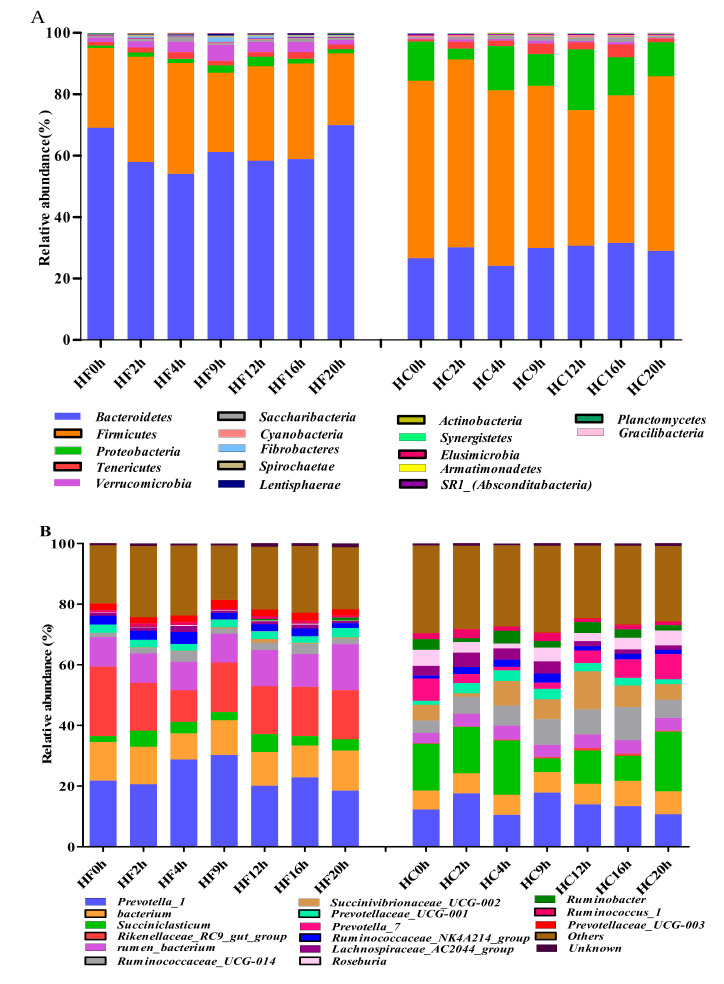
Histogram of relative abundance. The *x*-axis represents the groups, and the *y*-axis represents the relative abundance presented as percentage. (**A**) Relative abundance of the top 15 phyla. (**B**) Relative abundance of the top 15 genera. Only the top 15 species in abundance are shown in the figure; other species were combined as “others”.

At the phylum level, a total of 17 phyla were founded in this study; the dominant bacteria in the HF and HC treatments were *Bacteroidetes* (the average relative abundance of HF and HC were 64.57% and 25.36%, respectively) and followed by *Firmicutes*, *Proteobacteria*, *Tenericutes* and *Verrucomicrobia* (Figure 4A). The relative abundance of phylum *Bacteroidetes* and *Verrucomicrobia* were significantly higher in the HF treatment than that in the HC treatment. However, the relative abundance of phylum *Firmicutes* and *Proteobacteria* were significant higher in the HC treatment than that in the HF treatment (Table S3 in Supplementary Materials). In the HF dietary treatment, *Bacteroidetes* varied during the feeding cycle and was highest just before feeding and lowest approximately 4 h post-feeding; however, the *Verrucomicrobia* showed a reverse pattern, increased to the maximum value at 9 h post-feeding and then gradually increased to the initial value at approximately 20 h. In the HC dietary treatment, *Firmicutes* varied during the feeding cycle and was highest just before feeding and lowest approximately 12 h post-feeding.

At the genus level, the top 15 genera on the grounds of relative abundance of the rumen bacteria are displayed in Figure 4B. The relative abundance of *Rikenellaceae_RC9_gut_group*, *rumen_bacterium* and *Prevotellaceae_UCG-001* in the HF dietary treatment were significantly higher than that in the HC dietary treatment and significantly affected by the feed and time interaction (Table S4 in Supplementary Materials). Meanwhile, the relative abundance of *Prevotella_1*, belonging to the *Bacteroidetes*, in the HF dietary treatment was significantly higher than that in the HC dietary treatment and, at 9 h post-feeding time, was the highest and then gradually increased to the initial value. The relative abundance of *Succiniclasticum* and *Ruminococcus_1*, belonging to the *Firmicutes*, reached a maximum value at 2 h post-feeding, which could be due to the diet fermentation and provided enough energy for microbial growth (Table S4 in Supplementary Materials) and, in the HC dietary treatment, were significantly higher than that in the HF dietary treatment.

### 3.4. The Difference in the Microbial Composition Analysis and Functional Gene Prediction between the HF and HC Dietary Treatments

**Figure 5:**
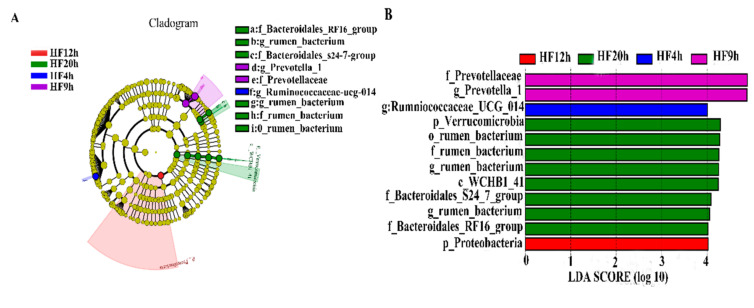

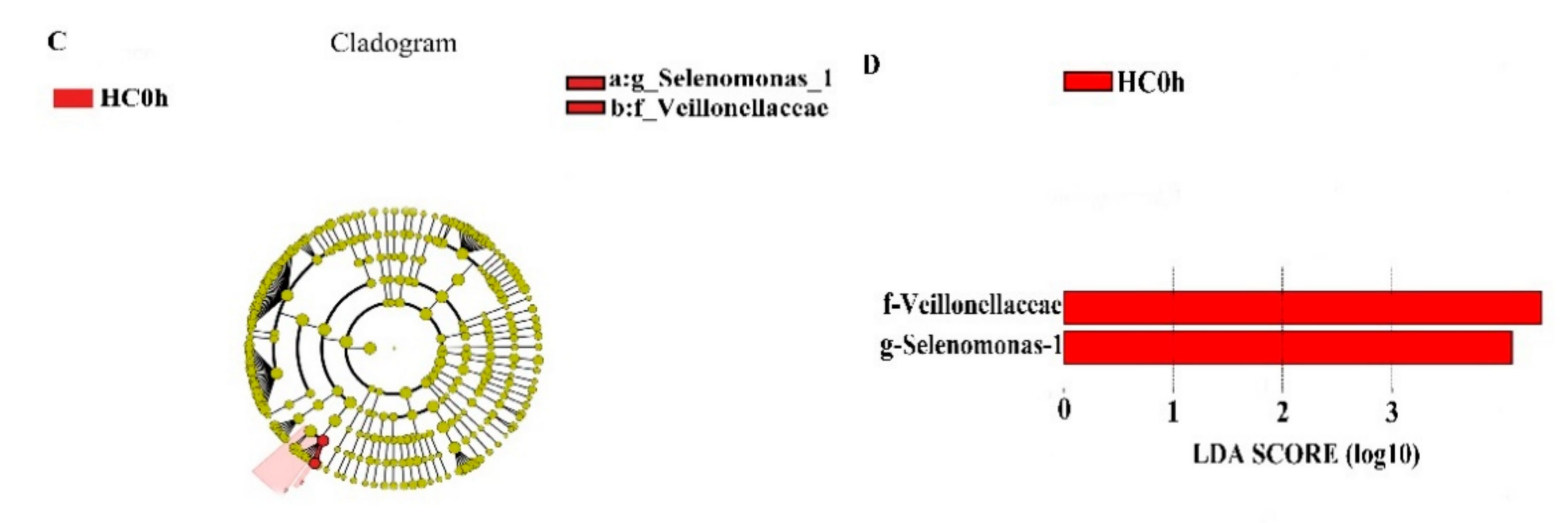
LefSe analysis. (**A**,**C**) The cladogram diagram shows the microbial species with significant differences in the HF (**A**) and HC (**C**) dietary treatments. The different colors indicate different groups, with the species classifications at the levels of phylum, class, order, family and genus shown from the inside to the outside. (**B**,**D**) Species with significant differences that have an linear discriminant analysis (LDA) score greater than the estimated value; the default score is 4.0 and 3.0 in the HF (**B**) and HC (**D**) dietary treatments, respectively. The length of the histogram represents the LDA score, i.e., the degree of influence of the species with the significant differences between the different groups.

LefSe analysis was conducted to reveal the significant ranking of the abundant modules. The cladogram (Figure 5A,C) showed differences of the intra-group of the the HF dietary treatment and the intra-group HC dietary treatment. The plot from the LefSe analysis (Figure 5B,D) displays the LDA scores of the microbial taxa, with significant differences of the intra-group of the HF dietary treatment and the intra-group of the HC dietary treatment. In the HF dietary treatment, the family *Bacteroidales_S24_7_group*, *rumen_bacterium* and *Bacteroidales_S24_7_group* and the phylum *Verrucomicrobia* were demonstrating significant differences between the HF20h (the group of the HF treatment at 20 h, the same as below) group and the other six groups; the genus *Ruminococcaceae_UCG_014* of the HF4h group was significantly higher than the other six groups; the biomarker showing significant differences between the HF9h group and the other six groups was *Prevotella_1*. In addition, the LefSe analysis showed that the phylum *Proteobacteria* of the HF12h group was significantly higher than that of the other six groups. In the HC dietary treatment, the biomarker showing significant differences between the HC0h group and the other six groups were the family *Veillonellaceae* and the genus *Selenomonas_1*.

**Figure 6:**
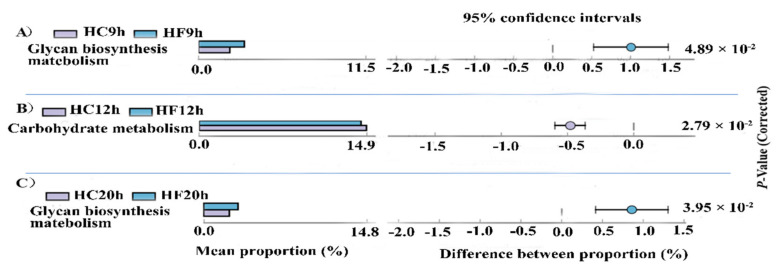
(**A**) The abundance ratio of glycan biosynthesis metabolism between the HF9handHC9h. (**B**) The abundance ratio of carbohydrate metabolism between the HF12handHC12h. (**C**) The abundance ratio of glycan biosynthesis metabolism between the HF20h and HC20h. Phylogenetic Investigation of Communities by Reconstruction of Unobserved States (PICRUSt) analysis. Variance analysis of the Kyoto Encyclopedia of Genes and Genomes (KEGG) metabolic pathways in the second level. The graphs show the abundance ratio of different functions in two groups of samples. The middle shows the difference between the proportions of functional abundance in the 95% confidence interval, and the value at the rightmost is the *p*-value. *p* < 0.05 represents the significant difference.

The species composition information obtained by comparing 16S sequencing data via PICRUSt software was used to infer the functional gene composition in the samples. By variance analysis of the Kyoto Encyclopedia of Genes and Genomes (KEGG) metabolic pathways, the differences and changes of the metabolic pathways of the functional genes in the microbiota between the samples of the different groups could be observed. During the feeding cycle, comparing the HF groups and HC groups, a total of three time points (9 h, 12 h and 20 h) showed significant differences of the carbohydrate metabolism (*p* < 0.05) (Figure 6). At 9 h and 20 h, the glycan biosynthesis and metabolism of the HF dietary treatment were significantly higher than that of the HC dietary treatment (*p* < 0.05) (Figure 6A,C). However, at 12 h, the carbohydrate metabolism of the HC dietary treatment was higher than that of the HF dietary treatment (*p* < 0.05) (Figure 6B), suggesting that the high-forage dietary can increase the glycan biosynthesis and metabolism.

## 4. Discussion

VFAs are the primary products of rumen fermentation in ruminants, which provide energy for the ruminant animals. During the feeding cycle, at 4 h post-feeding, the rumen pH declined its minimum value, while the TVFAs reached their maximum value. This result was consistent with Ash’s study [28]. At first, after 4 h post-feeding, the easily fermented carbohydrates, such as starch and soluble sugars, are rapidly fermented to produce VFAs; thus, the rumen TVFA content increased and was characterized by a decreased rumen pH. After 4 h post-feeding, the rumen pH gradually increased to the initial value, and the concentration of the TVFA gradually decreased to the initial value, which could be due to the VFA uptake by the host outpaced the VFA production from microbial fermentation. In the present study, the result showed that the TVFA was not affected by the dietary treatment, which was consistent with previous studies [29], but the HC treatment was higher than the HF treatment. The VFA absorption in the rumen is a passive process [28]; the VFAs transfer from the luminal to the surface of epithelial cells through the rumen movement, while the forage diets increase the rumen movement and decreases the rumen VFA of the HC dietary treatment. When the proportion of the forage diet increases, the proportion of the acetate rises and propionate decreases [30]. The Doyle et al. [31] research showed that, as the level of concentrate supplement in the lamb diet increased, the rumen pH value and the fiber degradation bacteria concentration decreased, but the TVFA concentration increased. When the diet changed from high-forage to high-concentrate, the molar ratio of acetate decreased, and the propionate and butyrate increased; the ratio of acetate:propionate was significantly reduced, changing the pattern of rumen fermentation [32], and previous studies suggested that fiber-degrading bacteria produced acetate and starch-decomposing bacteria produced propionate [33].

In this study, the changes of rumen microbiota of dairy cows fed two different diets during the feeding cycle were studied by high-throughput sequencing. The current research showed that the rumen microbiota diversity and bacterial species richness were significantly higher in the HF dietary treatment than that of the HC dietary treatment, which corresponded to the previous researches in dairy cows [29] and sheep [7]. The results indicated that the high-forage diet can increase the rumen microbiota diversity and could be due to the high-concentrate diet having low pH values inhibiting the growth of some acid-sensitive rumen bacteria.

The column diagram of relative abundance of the phylum showed that the highest relative content was *Bacteroidetes*, followed by *Firmicutes* and *Proteobacteria,* in the dairy cow rumen, regardless of the dietary and sampling time, which is similar to previous researches [34,35]. The phylum *Bacteroidetes* and *Firmicutes* were the two most relative abundant bacterial, which were able to degrade the complex plant polysaccharides and produce VFA [36]. Therefore, the phylum Bacteroidetes and Firmicutes varied and were consistent with the change of VFA during the feeding cycle.

In the present study, the relative abundance of *Firmicutes* in the dairy cow rumen of the HC dietary treatment was significantly higher than that of the HF dietary treatment. However, the results of this experiment are inconsistent with the previous results [37,38], which could be caused by the different animals or feeding environment. *Prevotella_1* was the most abundant bacterial genus and, under the HF dietary treatment, was higher than that under the HC dietary treatment and, at 9 h post-feeding, reached the maximum value. *Prevotella* was the dominant genus and more abundant under high-fiber diets, which have the capability to degrade hemicelluloses, pectin, starch or protein as energy sources [39,40]; therefore, *Prevotella* was affected by the energy and varied with the time after feeding. In this study, the genus *Succiniclasticum* was dominant within the phylum *Firmicutes*. *Succiniclasticum* specialized in fermenting succinate and converting it to propionate [35,38], which was consistent with the content of the propionate. During the feeding cycle, the *Firmicutes* variation was not significant, but the relative abundance of *Firmicutes* in the HF dietary treatment and HC dietary treatment decreased to the lowest values at 9 h and 12 h post-feeding, respectively. This phenomenon has been previously reported by Petri et al. [37]; the relative abundance of *Firmicutes* decreased at 4 h after the acidotic challenge; in this research, at 4 h post-feeding, the content of TVFA reached the maximum value, the pH reduced to the minimum value and the *Firmicutes* began to decrease since then. The relative abundance of *Firmicutes* decreased after the pH reduced to the minimum value and could be indicated that VFAs have a delayed effect on the phylum *Firmicutes*.

Notably, *Ruminobacter* and *Succinivibrionaceae_UCG-002* were dominant within the *Proteobacteria*. A previous study found that the *Ruminobacter* and *Succinivibrio* were found in high-grain diets [41]. The *Succinivibrionaceae* were positively correlated with the propionate, and the relative abundance of *Succinivibrio* was positively correlated with the butyrate [42], which was in agreement with the concentrations of propionate and butyrate, which were higher in the HF treatment than that of the HC treatment. The *Succinivibrio* genus has several species and strains—among which, *Succinivibrio dextrinosolvens* is the most well-studied—that produce succinate and convert it to propionate [35,41,43].

In the HF dietary groups, on the genus level, the *Ruminococcaceae_UCG-014* at 4 h post-feeding was significantly higher than the other groups by LefSe analysis. *Ruminococcaceae_UCG-014* was significantly positively correlated with the butyrate production [44]. On the contrary, in the HC dietary groups, at 0 h before feeding, the family *Veillonellaceae* and the genus *Selenomonas_1* were significantly higher than the other groups. *Veillonellaceae* has the ability to utilize lactate and pyruvate for acetate production via acetyl-CoA and butyrate production [45,46,47], suggesting that it would benefit from the highly fermentable diet, resulting in increased lactate concentrations in the rumen. *Selenomonas* can utilize lactate and carbohydrate to produce acetate, propionate and a small amount of butyrate and other metabolites, which also can promote bacteria growth and the prevention or treatment of rumen acidosis [48,49]. *Selenomonas ruminantium* could produce propionic acid through the succinate pathway (decarboxylation of succinate), which is known as a propionic acid-producing bacteria [50]. Hence, the increasing *Selenomonas* population could help utilize more fermentable substrates and lactates to maintain the ecological balance of the rumen microecosystem and reduce rumen acidosis in the high-concentrate feeding diet. Thus, the metabolism of the rumen in dairy cow is correlated with the existence of a major microbial type. Therefore, this study showed that the proportion and composition of the bacteria have changed considerably during the feeding cycle. The bacteria varied could be due to the rumen fermentation-changed energy supply and feed structure.

PICRUSt analysis indicated that in comparison of the same time points in two dietary treatments, the abundance of functional genes in the glycan biosynthesis and metabolism pathways was significantly higher in the HC dietary treatment than that of the HF dietary treatment at 9 and 12 h post-feeding. This indicates that the contents of the soluble carbohydrates were higher in the HC dietary treatment than that of the HF dietary treatment. In the present study, the CMCase and β-glucosidase activity of the HC dietary treatment were significantly higher than that of the HF dietary treatment at 9 h and 12 h poet-feeding (Figure S1 in Supplementary Materials). Consequently, it demonstrated that the glycan biosynthesis and metabolism pathways were significantly higher in the HC dietary treatment than that of the HF dietary treatment at 9 and 12 h post-feeding. However, the carbohydrate metabolism pathways were significantly higher in the HF dietary treatment than that of the HC dietary treatment. This indicated that the structural carbohydrates metabolism was higher in the HF20h group than that of the HC20h group, and the forage diet increased the fiber metabolism. However, PICRUSt is just a way to predict the functional genes. Hence, the accuracy of the gene function information was confirmed by metagenomics.

## 5. Conclusions

In conclusion, the VFA concentration decreased, and the ruminal pH increased in the HF dietary treatment compared with those in the HC dietary treatment. The 4 h post-feeding was a turning point of the rumen fermentation during the feeding cycle. However, the microbial diversity increased in the HF dietary treatment compared with that in the HC dietary treatment. The phyla *Firmicutes*, *Bacteroidetes* and *Proteobacteria* and the genus *Prevotella* were the dominant bacterial, and the changes in response to the changes of diets and post-feeding times indicated that the cow rumen microbiome were structurally similar but compositionally distinct during the feeding cycle. With the increasing amount of dietary forage, the fiber metabolism was increased after 9 h post-feeding. The results of this study provided a better understanding of how the bacterial ecosystem variation during the feeding cycle of cows were fed the high-forage/concentrate diets.

## Figures and Tables

**Table 1:** Rumen pH over the course of a feeding cycle in dairy cows receiving two diets: high-forage (HF) and high-concentrate (HC).

HF							HC						
Time	Observed pH			C (H+)			Observed pH				C (H+)		
0 h	6.79	6.92	6.78	1.62 × 10^−7^	1.20 × 10^−7^	1.66 × 10^−7^	6.94		6.9	6.85	1.15 × 10^−7^	1.26 × 10^−7^	1.41 × 10^−7^
2 h	6.52	6.52	6.43	3.02 × 10^−7^	3.02 × 10^−7^	3.71 × 10^−7^	6.54		6.48	6.35	2.88 × 10^−7^	3.31 × 10^−7^	4.47 × 10^−7^
4 h	6.1	6.02	6.15	7.94 × 10^−7^	9.55 × 10^−7^	7.08 × 10^−7^	5.85		5.61	5.73	1.41 × 10^−6^	2.45 × 10^−6^	1.86 × 10^−6^
6 h	6.38	6.12	6.28	4.17 × 10^−7^	7.58 × 10^−7^	5.25 × 10^−7^	5.61		5.5	5.62	2.45 × 10^−6^	3.16 × 10^−6^	2.40 × 10^−6^
9 h	6.47	6.17	6.31	3.39 × 10^−7^	6.76 × 10^−7^	4.90 × 10^−7^	5.88		5.64	5.74	1.32 × 10^−6^	2.29 × 10^−6^	1.82 × 10^−6^
12 h	6.53	6.31	6.41	2.95 × 10^−7^	4.90 × 10^−7^	3.89 × 10^−7^	6.12		6.07	5.94	7.59 × 10^−7^	8.51 × 10^−7^	1.15 × 10^−6^
16 h	6.69	6.6	6.65	2.04 × 10^−7^	2.51 × 10^−7^	2.24 × 10^−7^	6.43		6.39	6.35	3.72 × 10^−7^	4.07 × 10^−7^	4.47 × 10^−7^
20 h	6.79	6.68	6.68	1.62 × 10^−7^	2.09 × 10^−7^	2.09 × 10^−7^	6.58		6.61	6.55	2.63 × 10^−7^	2.45 × 10^−7^	2.82 × 10^−7^
24 h	6.92	6.78	6.79	1.20 × 10^−7^	1.66 × 10^−7^	1.62 × 10^−7^	6.88		6.83	6.74	1.32 × 10^−7^	1.48 × 10^−7^	1.82 × 10^−7^
**Item**	**Diet**	**Time**								**SEM**	***p*-Values**		
		**0 h**	**2 h**	**4 h**	**9 h**	**12** h	**16 h**	**20 h**	**24 h**		**Feed**	**Time**	Feed * Time
C(H+)	HF	1.50 × 10^−7 e^	3.25 × 10^−7 cd^	8.19 × 10^−7 Ba^	5.02 × 10^−7 Bb^	3.91 × 10^−7 Bbc^	2.26 × 10^−7 Bde^	1.93 × 10^−7 Bde^	1.50 × 10^−7 e^	0.000	<0.0001	<0.0001	<0.0001
	HC	1.27 × 10^−7 c^	3.55 × 10^−7 c^	1.91 × 10^−6 Aa^	1.81 × 10^−6 Aa^	9.19 × 10^−7 Ac^	4.09 × 10^−7 Ac^	2.63 × 10^−7 Ac^	1.54 × 10^−7 c^				
pH	HF	6.83	6.49	6.09	6.30	6.41	6.65	6.71	6.83				
	HC	6.90	6.45	5.72	5.74	6.04	6.39	6.58	6.81				

(1) C (H+): Hydrogen ion concentration. (2.) A,B means in the same column followed by different letters show treatments (HF and HC) differ (*p* < 0.05). a−e means in the same row followed by different letters show times differ (*p* < 0.05).

**Table 2:** Rumen fermentation parameters over the course of a feeding cycle in dairy cows receiving two diets: HF and HC.

Item	Diet	After Feed Time (h)							SEM	*p*-Values		
		0 h	2 h	4 h	9 h	12 h	16 h	20 h		Feed	Time	Feed * Time
TVFA ^a^, mM	HF	106.74 ^d^	125.88 ^cd^	155.38 ^a^	147.09 ^ab^	128.65 ^bc^	110.29 ^cd^	116.83 ^cd^	6.5148	0.167	<0.001	0.174
	HC	118.35 ^b^	137.47 ^b^	164.60 ^a^	159.40 ^a^	135.67 ^b^	133.04 ^b^	125.57 ^b^				
VFA ^b^, molar % of TVFA												
Acetate	HF	69.75 ^Abc^	70.69 ^Abc^	73.65 ^Aab^	76.38 ^Aa^	71.96 ^Ab^	67.20 ^Ac^	67.54 ^Ac^	0.770	<0.001	<0.001	<0.001
	HC	61.54 ^Ba^	57.46 ^Bb^	57.28 ^Bb^	53.73 ^Bd^	54.80 ^Bcd^	54.54 ^Bcd^	56.25 ^Bbc^				
Propionate	HF	16.09 ^Babc^	16.35 ^Babc^	14.39 ^Bc^	14.47 ^Bbc^	17.08 ^Bab^	18.37 ^Ba^	18.48 ^Ba^	0.576	<0.001	<0.001	<0.001
	HC	24.98 ^Ad^	27.86 ^Abc^	27.66 ^Ac^	29.59 ^Aa^	29.54 ^Aa^	29.03 ^Aa^	28.82 ^Aab^				
Butyrate	HF	7.82 ^Bc^	7.83 ^Bc^	8.04 ^Bbc^	5.84 ^Bd^	7.50 ^Bc^	10.16 ^Ba^	9.112 ^Bab^	0.557	<0.001	0.001	<0.001
	HC	9.17 ^Ad^	10.35 ^Ad^	11.19 ^Ac^	13.17 ^Aa^	12.51 ^Ab^	12.56 ^Ab^	11.13 ^Ac^				
Isobutyrate	HF	4.85 ^Aa^	3.75 ^Ab^	2.89 ^Ade^	2.42 ^Ae^	2.50 ^Ae^	3.19 ^Acd^	3.65 ^Abc^	0.274	<0.001	<0.001	0.008
	HC	1.09 ^Ba^	0.94 ^Bb^	0.81 ^Bc^	0.72 ^Bd^	0.69 ^Bd^	0.86 ^Bc^	0.88 ^Bbc^				
Valerate	HF	0.50 ^Bb^	0.62 ^Ba^	0.46 ^Bb^	0.40 ^Bb^	0.46 ^Bb^	0.45 ^Bb^	0.44 ^Bb^	0.054	<0.001	<0.001	0.090
	HC	1.74 ^Ab^	1.91 ^Aa^	1.78 ^Aab^	1.56 ^Acd^	1.49 ^Ad^	1.79 ^Aab^	1.66 ^Abc^				
Isovalerate	HF	1.00 ^Ba^	0.76 ^Bb^	0.56 ^Bcd^	0.49 ^Bd^	0.50 ^Bd^	0.64 ^Bbc^	0.76 ^Bb^	0.049	<0.001	<0.001	0.018
	HC	1.48 ^Aa^	1.46 ^Aa^	1.27 ^Ab^	1.22 ^Ab^	0.97 ^Ac^	1.21 ^Ab^	1.26 ^Ab^				
A:P ^c^	HF	4.4 ^Ab^	4.41 ^Ab^	5.12 ^Aa^	5.31 ^Aa^	4.22 ^Abc^	3.69 ^Ac^	3.66 ^Ac^	0.172	<0.001	<0.001	<0.001
	HC	2.46 ^Ba^	2.06 ^Bb^	2.07 ^Bb^	1.82 ^Bd^	1.85 ^Bcd^	1.88 ^Bcd^	1.95 ^Bbc^				

(1) ^a^ TVFA: total volatile fatty acids, including acetate, propionate, butyrate, isobutyrate, valerate and isovalerate. ^b^ VFA: volatile fatty acids. ^c^ A:P: acetate:propionate. (2) A,B means in the same column followed by different letters show treatments (HF and HC) differ (*p* < 0.05). a−e means in the same row followed by different letters show times differ (*p* < 0.05).

**Table 3:** Alpha-diversity indices in the rumen microbiota of dairy cows receiving two diets: HF and HC ^1^.

Sample		After Feed Time (h)							SEM	*p*-Values		
		0 h	2 h	4 h	9 h	12 h	16 h	20 h		Feed	Time	Feed * Time
OTU	HF	1291 ^Ad^	1304 ^Ad^	1348 ^Ac^	1396 ^Aab^	1420 ^Aa^	1371 ^Abc^	1353 ^Ac^	18.19	<0.001	<0.001	0.228
	HC	1032 ^Bc^	1031 ^Bc^	1013 ^Bc^	1070 ^Bbc^	1186 ^Ba^	1153 ^Bab^	1097 ^Babc^				
ACE	HF	1116 ^bc^	1106 ^c^	1131 ^Aabc^	1147 ^ab^	1169 ^Ba^	1139 ^Babc^	1133 ^Babc^	19.59	<0.001	0.003	0.174
	HC	1110 ^c^	1083 ^c^	1079 ^Bc^	1153 ^bc^	1327 ^Aa^	1294 ^Aab^	1190 ^Aabc^				
Chao 1	HF	1126 ^bc^	1112 ^c^	1145 ^Abc^	1157 ^ab^	1193 ^Ba^	1147 ^Bbc^	1142 ^bc^	21.04	<0.001	0.012	0.223
	HC	1131 ^c^	1094 ^c^	1093 ^Bc^	1177 ^bc^	1377 ^Aa^	1331 ^Aab^	1222 ^abc^				
Shannon	HF	5.51 ^Abc^	5.55 ^abc^	5.45 ^Ac^	5.52 ^bc^	5.66 ^Aa^	5.59 ^Aab^	5.66 ^Aa^	0.07	<0.001	0.315	0.273
	HC	4.88 ^Bb^	5.23 ^ab^	5.01 ^Bab^	5.34 ^a^	5.05 ^Bab^	5.23 ^Bab^	4.90 ^Bb^				
Simpson	HF	0.011 ^Bab^	0.011 ^Bab^	0.011 ^Ba^	0.011 ^ab^	0.009 ^Bbc^	0.010 ^abc^	0.009 ^Bc^	0.0061	<0.001	0.018	0.600
	HC	0.030 ^A^	0.033 ^A^	0.029 ^A^	0.016	0.026 ^A^	0.021	0.035 ^A^				

(1) The operational taxonomic units (OTUs) were defined with 97% similarity. The richness estimators (ACE and Chao 1) and diversity indices (Shannon and Simpson) were calculated. (2) A,B means in the same column followed by different letters show treatments (HF and HC) differ (*p* < 0.05). a−d means in the same row followed by different letters show times differ (*p* < 0.05).

## Data Availability

The Illumina sequencing raw data were deposited in the NCBI server (https://www.ncbi.nlm.nih.gov/sra/?term=SRP141142) and are available to the public (BioProject ID: PRJNA45088 and SRX3971715 to SRX3971756).

## Supplementary Materials

The following are available online at https://www.mdpi.com/2076-2615/10/6/957/s1. Table S1: Ingredients and nutritional composition of experimental diets. (Dry matter basis, %), Table S2: The Evaluation results of each sequencing data, Table S3: Comparison of the dominant phylum (average relative abundance ≥1% for at least one treatment) within the rumen, Table S4: Comparison of the dominant genus (average relative abundance ≥1% for at least one treatment) within the rumen, Figure S1: Xylanase, CMCase and β-glucosidase activity dynamics during the feeding cycle. Red line represent for HF dietary treatment, black line represent for HC dietary treatment.
