# Supplementary material for: The Effects of Different Concentrate-to-Forage Ratio Diets on Rumen Bacterial Microbiota and the Structures of Holstein Cows during the Feeding Cycle
Source: Animals (Basel). 2020 May 31;10(6):957. doi: 10.3390/ani10060957 (PMC7341334; doi:10.3390/ani10060957)
Supplement: Supplementary file 1 [file animals-10-00957-s001.pdf]

The Effect of High Concentrate/Forage Diet on Rumen Bacterial Microbiota and structure of Holstein Cows during the feeding cycle

Lijun Wang [1], Yang Li[2], Yonggen Zhang [2*] and Lihua Wang[1*]

[1] College of Animal Science and Technology, Qingdao agricultural university, No. 700 of Changcheng Road, Qingdao 266000, China; wlj880626@163.

[2] College of Animal Science and Technology, Northeast Agricultural University, No. 600 of Changjiang Road, Harbin 150030, China; dahai0806702@126.com.

* Correspondence: zhangyonggen409@126.com; Tel./Fax: +86-451-5519-0840; lhwang2007@163.com.

Table S1 Ingredients and nutritional composition of experimental diets. (Dry matter basis, %)

**Item**	**HF[3] diet**	**HC[4] diet**
Ingredient, % of DM		
Ensiled maize stalks	57.0	19.0
Chinese ryegrass hay	4.0	6.0
Alfalfa pellets	9.0	5.0
Steam-flaked maize	13.0	54.0
Soybean curb residue	15.2	13.7
Mineral mix[1]	1.8	2.3
**Nutrition composition**		
DM	67.8	45.5
ME[2] (MJ/kg)	9.07	11.70
CP, % of DM	12.7	12.7
NDF, % of DM	54.2	35.3
ADF, % of DM	25.3	19.2
Starch, % of DM	12.2	40.9
Ca, % of DM	0.64	0.63
P, % of DM	0.32	0.33

[1] Mineral mix contained 18.50% Ca, 6.00% P, 4.2% Mg, 1.4% K, 2.6% S, 7.5% Na, 12.0% Cl, 30 mg/kg of Se, 0.25% Zn, 0.25% Fe, 0.25% Mn, 1,100 mg/kg of Cu, 15 mg/kg of I, 265,000 IU/kg of vitamin A, 110,200 IU/kg of vitamin D, and 2,300 IU/kg of vitamin K.

[2] ME, metabolizable energy; CP, crude protein; NDF, neutral detergent fiber; ADF, acid detergent fiber.

[3] HF diet contained 70% forage; [4] HC diet contained 30% forage.

Table S2 The Evaluation results of each sequencing data.

Sample ID	PE Reads	Raw Tags	Clean Tags	Effective Tags	AvgLen(bp)	GC(%)	Q20(%)	Effective(%)
A01	80,085	75,336	63,946	63,329	420	54.54	97.14	79.08
A02	73,309	68,998	58,478	57,756	421	54.39	97.04	78.78
A03	74,530	70,101	58,952	58,457	421	54.7	97.09	78.43
A21	75,247	71,045	60,269	59,639	421	54.45	97.14	79.26
A22	80,060	75,710	64,712	64,611	418	54.14	97.31	80.7
A23	79,991	75,034	63,241	62,601	421	54.23	97.18	78.26
A41	80,064	75,701	64,196	63,846	420	53.99	97.2	79.74
A42	79,708	75,939	64,814	64,351	420	54.07	97.24	80.73
A43	80,000	75,027	64,042	63,615	418	53.75	97.21	79.52
A91	79,736	74,218	62,330	61,824	422	54.11	96.96	77.54
A92	79,798	74,779	62,863	62,425	421	54.09	97.03	78.23
A93	80,015	75,366	64,062	63,404	421	54.07	97.08	79.24
A121	80,224	75,438	63,881	63,270	422	54.23	97.04	78.87
A122	80,264	75,647	64,609	63,797	420	54.2	97.23	79.48
A123	79,988	74,617	63,266	62,625	420	54.12	97.11	78.29
A161	80,090	75,607	64,284	63,798	421	54.06	97.09	79.66
A162	79,892	75,184	64,302	63,781	420	53.92	97.16	79.83
A163	80,043	75,293	64,677	63,847	419	54.15	97.15	79.77
A201	80,118	75,172	63,707	63,233	422	54.37	97.03	78.92
A202	79,716	75,112	63,963	63,543	422	54.29	97.13	79.71
A203	79,978	75,477	64,621	63,809	420	54.39	97.18	79.78
S01	80,100	74,852	63,907	61,695	417	53.34	97.5	77.02
S02	79,850	74,757	63,924	62,701	420	53.31	97.32	78.52
S03	79,854	75,785	65,142	63,627	419	53.41	97.44	79.68
S21	80,085	74,935	63,983	63,141	419	53.79	97.36	78.84
S22	79,720	75,358	64,784	64,377	415	52.75	97.37	80.75
S23	80,099	75,476	64,472	63,506	419	53.14	97.24	79.28
S41	79,835	75,530	64,952	63,830	417	53.56	97.42	79.95
S42	80,126	75,672	64,674	63,656	420	53.19	97.31	79.44
S43	79,992	75,521	64,289	63,486	420	53.42	97.4	79.37
S91	80,048	75,749	65,339	64,584	416	52.8	97.36	80.68
S92	76,324	72,979	63,625	63,203	414	52.55	97.52	82.81
S93	79,897	74,615	63,165	62,946	420	52.17	97.2	78.78
S121	80,026	74,724	63,274	62,292	420	53.38	97.2	77.84
S122	79,852	74,838	63,766	62,981	420	53.02	97.18	78.87
S123	80,176	75,643	64,813	64,584	420	52.11	97.26	80.55
S161	80,197	75,239	64,151	63,291	419	53.44	97.24	78.92
S162	79,871	75,494	64,712	64,505	420	52.19	97.26	80.76
S163	79,752	75,090	64,604	64,398	416	52.13	97.32	80.75
S201	79,920	75,189	63,823	62,963	420	53.66	97.29	78.78
S202	79,743	74,437	63,579	62,470	419	53.28	97.24	78.34
S203	80,067	74,618	63,261	61,996	420	53.46	97.17	77.43

Note: PE Reads were the double-ended reads; Raw Tags were spliced ofthe double-ended reads

Table S3 Comparison of the dominant phylum (average relative abundance ≥1% for at least one treatment) within the rumen.

**Phylum**	**Feed**	**After feeding time (h)**							**SEM**	***P*-Value**		
		**0 h**	**2 h**	**4 h**	**9 h**	**12 h**	**16 h**	**20 h**		**feed**	**time**	**feed×time**
Bacteroidetes	HF	69.25	59.89	54.12	61.17	58.60	58.92	69.98	16.11	<.0001	0.3768	0.4942
	HC	26.57	30.46	24.14	30.01	30.64	31.60	29.04				
Firmicutes	HF	25.88	32.43	36.09	23.36	30.52	31.08	25.87	27.19	<.0001	0.5527	0.5926
	HC	57.95	60.92	57.06	52.54	44.38	48.20	56.86				
Proteobacteria	HF	0.78	1.36	1.37	1.32	3.08	1.54	2.40	1.4980	<.0001	<.0001	0.0009
	HC	12.59	3.56	14.50	10.50	19.69	12.42	11.07				
Tenericutes	HF	1.12	1.52	2.14	1.58	1.55	2.10	1.47	0.1791	0.0272	0.0027	0.0723
	HC	0.76	2.19	1.81	3.40	2.22	4.05	1.17				
Verrucomicrobia	HF	1.37	2.09	3.45	1.46	3.29	3.35	5.17	0.0583	<.0001	<.0001	<.0001
	HC	0.32	0.72	0.47	0.93	0.48	0.80	0.14				
Saccharibacteria	HF	0.52	0.63	1.62	0.61	0.72	1.14	0.56	0.0739	0.2022	0.0328	0.4525
	HC	0.42	0.81	1.13	1.40	1.25	1.59	0.68				
Cyanobacteria	HF	0.14	0.19	0.12	0.14	0.24	0.15	0.30	0.0058	<.0001	0.1530	0.2047
	HC	0.48	0.65	0.28	0.59	0.61	0.64	0.41				
Fibrobacteres	HF	0.04	0.48	0.11	0.14	0.71	0.33	1.37	0.0080	<.0001	<.0001	<.0001
	HC	0.07	0.08	0.00	0.01	0.01	0.02	0.03				
SR1_Abs-	HF	0.23	0.24	0.23	0.20	0.17	0.23	0.19	0.0129	0.0082	0.7856	0.8057
conditabacteria	HC	0.66	0.37	0.27	0.34	0.45	0.41	0.34				
Spirochaetae	HF	0.18	0.57	0.17	0.55	0.50	0.52	0.60	0.0048	<.0001	0.0184	0.0372
	LF	0.04	0.09	0.01	0.02	0.02	0.02	0.03				
Lentisphaerae	HF	0.25	0.31	0.30	0.43	0.38	0.44	0.62	0.0034	<.0001	0.1318	0.1459
	LF	0.0000	0.0048	0.0006	0.0006	0.0090	0.0063	0.0042				

Actinobacteria	HF	0.14	0.09	0.11	0.10	0.04	0.05	0.03	0.0012	0.2188	0.1426	0.1838
	LF	0.03	0.07	0.20	0.16	0.09	0.11	0.09				
Synergistetes	HF	0.054	0.066	0.091	0.061	0.065	0.056	0.065	0.0001	<.0001	0.0015	0.0138
	LF	0.088	0.047	0.121	0.073	0.131	0.108	0.128				
Elusimicrobia	HF	0.033	0.074	0.037	0.030	0.085	0.041	0.173	0.0001	<.0001	<.0001	<.0001
	LF	0.002	0.008	0.001	0.003	0.003	0.007	0.003				
Armatimonadetes	HF	0.010	0.022	0.023	0.012	0.008	0.017	0.014	0.0000	<.0001	0.5141	0.6237
	LF	0.000	0.000	0.001	0.000	0.000	0.000	0.000				
Planctomycetes	HF	0.002	0.032	0.008	0.019	0.011	0.013	0.021	0.0000	<.0001	0.1896	0.1685
	LF	0.000	0.000	0.000	0.000	0.002	0.000	0.000				
Gracilibacteria	HF	0.002	0.004	0.003	0.001	0.006	0.004	0.001	0.0000	<0001	0.1954	0.3508
	LF	0.008	0.016	0.003	0.012	0.013	0.014	0.013				

a, b, c, and d Values in the same row with different superscripts differ significantly (P <0.05).

Table S4 Comparison of the dominant genus (average relative abundance ≥1% for at least one treatment) within the rumen.

	Genera	Feed	After feeding time (h)							SEM	*P*-Value²		
			0 h	2 h	4 h	9 h	12 h	16 h	20 h		Feed	Time	Feed×Time
Bacteroidetes	*Prevotella_1*	HF	21.88	21.53	28.75	30.28	20.11	22.84	18.48	5.264	<.001	0.034	0.162
		HC	12.44	17.91	10.55	17.89	13.91	13.43	10.75				
	bacterium	HF	12.75	12.37	8.64	11.46	11.14	10.51	13.23	0.492	<.001	0.064	0.045
		HC	6.21	6.57	6.61	6.80	6.79	8.28	7.58				
	Rikenellaceae_RC9_gut_group	HF	22.90	16.44	10.48	16.30	15.93	16.31	16.18	1.108	<.001	0.002	0.002
		HC	0.21	0.17	0.28	0.49	0.79	0.73	0.32				
	rumen_bacterium	HF	9.68	9.85	9.39	9.53	11.99	10.86	15.16	0.593	<.001	0.014	0.030
		HC	3.33	4.20	4.61	3.97	4.45	4.41	4.23				
	Prevotellaceae_UCG-001	HF	2.73	2.51	2.19	2.56	2.53	2.20	3.08	0.094	0.651	0.074	0.001
		HC	1.30	3.32	3.50	3.41	2.71	2.52	1.63				
	Prevotella_7	HF	0.00	0.00	0.01	0.05	0.08	0.02	0.01	0.715	<.001	0.005	0.005
		HC	7.25	3.02b	1.20	2.09	4.15	6.06	8.3				
	Prevotellaceae_UCG-003	HF	2.25	2.06	2.25	2.94	2.18	2.66	2.12	0.027	<.001	0.135	0.208
		HC	0.16	0.17	0.08	0.21	0.30	0.24	0.18				
Firmicutes	*Ruminococcaceae_UCG-014*	HF	1.39	1.68	3.44	1.70	2.38	3.33	1.80	0.839	<.001	0.006	0.173
		HC	4.04	5.57	6.64	8.50	8.16	10.82	5.94				
	Ruminococcaceae_NK4A214_group	HF	2.86	2.85	3.99	2.11	2.25	2.54	1.61	0.192	0.014	0.042	0.143
		HC	0.96	2.39	2.34	2.99	1.41	1.82	1.44				
	Lachnospiraceae_AC2044_group	HF	1.04	1.00	1.98	0.62	0.99	1.28	0.72	0.307	<.001	0.033	0.056
		HC	3.24	4.67	3.70	3.99	1.69	1.49	1.49				
	Succiniclasticum	HF	1.94	4.73	3.81	2.69	5.81	3.11	3.77	3.610	<.001	0.008	0.017

		HC	15.37	15.33	17.90	4.51	11.17	8.51	19.66				
	Roseburia	HF	0.12	0.15	0.27	0.14	0.25	0.25	0.14	0.710	<.001	0.554	0.453
		HC	5.25	3.40	1.63	4.46	2.71	3.80	4.85				
	Ruminococcus_1	HF	0.69	1.04	0.91	0.53	0.91	0.91	0.65	0.071	<.001	0.012	0.010
		HC	1.88	2.92	1.33	2.60	0.97	1.40	1.06				
	Christensenellaceae_ R-7_group	HF	1.68	1.85	2.11	1.11	1.34	1.30	1.02	0.044	0.001	0.015	0.121
		HC	0.44	1.16	1.31	1.33	0.70	0.84	0.72				
Proteobacteria	*Ruminobacter*	HF	0.02	0.09	0.03	0.07	0.51	0.08^{c}	0.97	0.350	<.001	0.316	0.153
		HC	3.54	1.25	4.19	2.19	3.63	2.72	1.77				
	Succinivibrionaceae_UCG-002	HF	0.05	0.24	0.15	0.43	1.22	0.31	0.38	2.664	<.001	0.088	0.211
		HC	5.17	1.13	8.10	6.66	12.45	7.08	5.12				

a, b, c Values in the same row with different superscripts differ significantly (P <0.05).

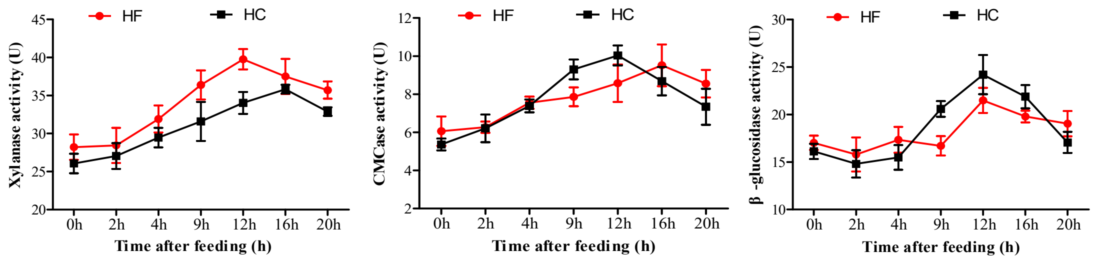

Fig.S1 Xylanase, CMCase and β-glucosidase activity dynamics during the feeding cycle. Red line represent for HF dietary treatment, black line represent for HC dietary treatment.
